# Case Report: Diagnostic and therapeutic challenges in acute myocardial infarction years after aortic replacement surgery: a case of severe vascular tortuosity

**DOI:** 10.3389/fcvm.2025.1611019

**Published:** 2025-06-05

**Authors:** Yuanzhen Su, Gaixia Zhai, Lei Zhang, Hongliang Tian

**Affiliations:** ^1^ Department of Cardiology, Zibo Central Hospital, Zibo, China; ^2^ Department of Ophthalmology, Zibo Central Hospital, Zibo, China

**Keywords:** Stanford type A aortic dissection, aortic computed tomography angiography, coronary artery disease, acute myocardial infarction, coronary angiography

## Abstract

This study delineates the diagnostic and therapeutic strategies for acute myocardial infarction occurring years after stent implantation for Stanford type A aortic dissection. Emergency coronary angiography presented substantial technical challenges attributable to the lack of recent aortic imaging data and marked tortuosity of the brachiocephalic trunk resulting from postoperative anatomical changes. Consequently, while selective left coronary angiography was successfully completed, visualization of the right coronary artery necessitated non-selective contrast administration via a pigtail catheter. This case underscores the pivotal role of preoperative aortic computed tomography angiography (CTA) in hemodynamically stable patients, as it provides essential vascular anatomical information that may circumvent procedural complexities during coronary angiography. Building upon these observations, we advocate an “aorto-coronary combined assessment” strategy for post-aortic surgery patients, integrating systematic imaging surveillance to facilitate early identification of coronary lesions. Such an approach permits the timely implementation of intensive medical therapy or elective revascularization, thereby mitigating the risk of acute cardiovascular events.

## 1 Introduction

Stanford type A aortic dissection constitutes one of the most life-threatening emergencies in cardiovascular medicine, characterized by blood entry into the aortic media through an intimal tear, resulting in false lumen formation with frequent involvement of the ascending aorta (1, 2). Epidemiological data reveal a time-dependent mortality pattern in untreated patients, demonstrating an incremental risk of 1%–2% per hour and reaching a cumulative 48-hour mortality rate of 50% (3). Although emergent surgical intervention significantly improves clinical outcomes (4), patients remain at risk for various postoperative complications (5–7), necessitating long-term antihypertensive therapy and systematic imaging surveillance. Current clinical practice predominantly emphasizes aortic pathology evaluation, frequently neglecting concurrent coronary artery disease assessment. Published evidence indicates that approximately 34.8% of patients with acute type A dissection present with >50% coronary stenosis (8), a finding corroborated by Larson and Edwards' case series, which identified severe coronary artery disease in 22% of 121 dissection cases (9). Coronary heart disease arises from a combination of modifiable and non-modifiable risk factors, including advanced age, male sex, genetic predisposition dyslipidemia, hypertension, smoking, diabetes mellitus, obesity, sedentary lifestyle, and chronic inflammation. Coronary angiography in post-aortic replacement patients presents unique technical challenges, including: (1) postoperative anatomical alterations, (2) increased procedural complexity, (3) comorbid condition management, and (4) imaging interpretation difficulties.

## 2 Case report

A 55-year-old male was admitted with persistent precordial pain lasting 22 h. Physical examination: Blood pressure 141/88 mmHg, heart rate 82 beats per minute, respiratory rate 20 breaths per minute. No significant abnormalities were detected on cardiopulmonary auscultation. Electrocardiographic findings demonstrated sinus rhythm with pathological Q waves and ST-segment elevation in leads III and aVF. Serum cardiac biomarkers were significantly elevated, with troponin I at 3.69 ng/ml, CK-MB at 10 ng/ml, and myoglobin at 57.9 ng/ml, while D-dimer levels and coagulation profile remained within normal limits. The patient's medical history was significant for: (1) severe systemic hypertension (peak systolic blood pressure: 200 mmHg), (2) surgically repaired Stanford type A aortic dissection (aortic replacement with stent implantation performed four years prior), and (3) stable follow-up aortic computed tomography angiography (CTA) findings three months before the current admission. Final diagnoses included: (1) acute ST-segment elevation myocardial infarction (inferior wall) secondary to coronary artery disease (Killip class I), (2) stage 3 hypertension (very high-risk category), and (3) status post surgical repair of type A aortic dissection.

**Figure 1:**
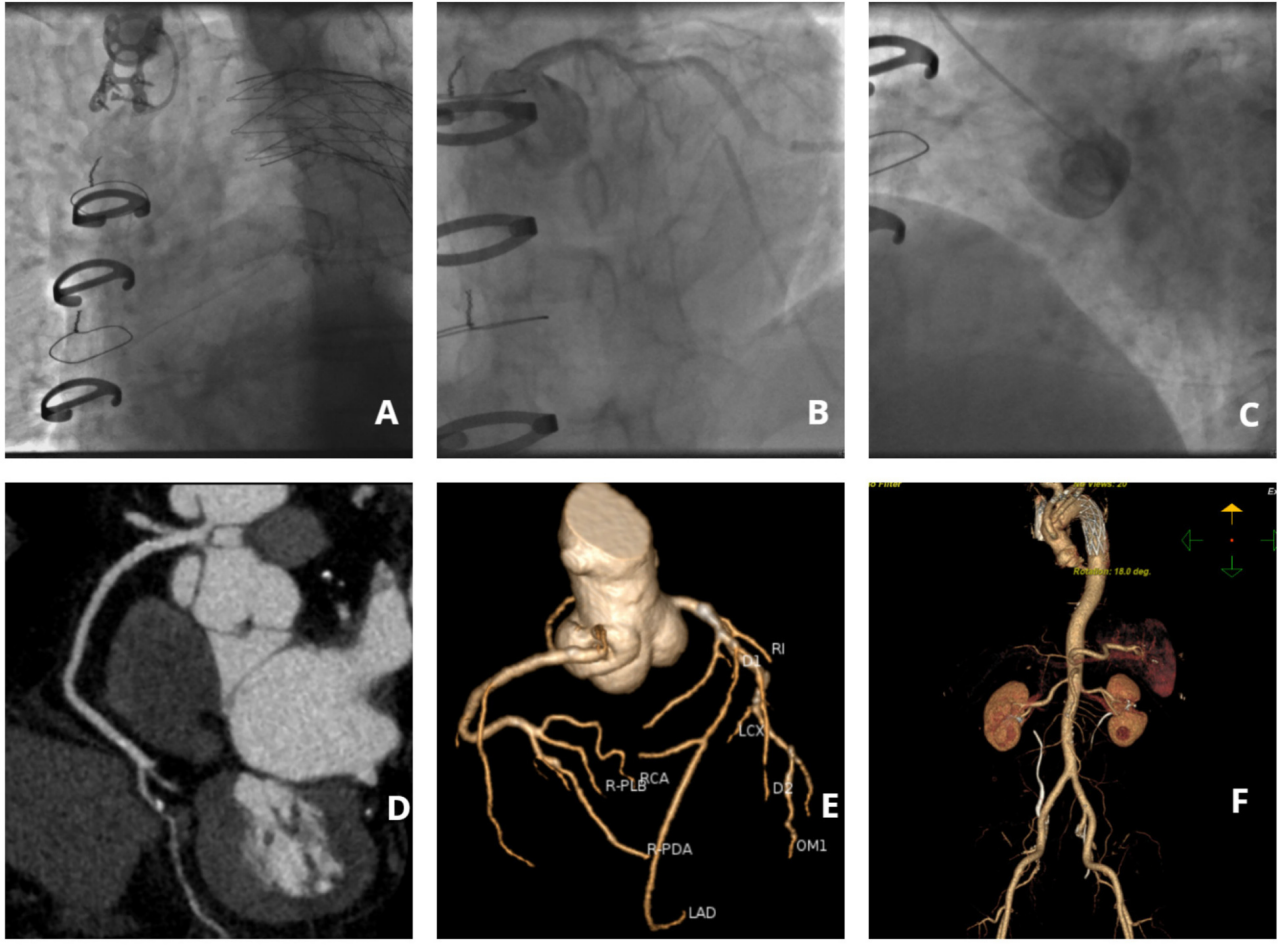
Imaging findings of coronary and aortic vasculature. **(A)** Angiographic visualization of brachiocephalic trunk tortuosity; **(B)** Selective angiography of the left coronary artery; **(C)** Non-selective right coronary angiography performed via pigtail catheter; **(D)** Computed tomography angiography (CTA) of the right coronary artery; **(E)** Coronary CTA; **(F)** Aortic CTA.

Following a comprehensive diagnostic evaluation excluding aortic dissection recurrence, emergency coronary angiography was performed after obtaining informed consent within 60 min of hospital admission. Due to the patient's complex vascular surgical history and absence of recent aortic imaging studies, right radial arterial access was strategically selected to minimize procedural risks. The angiographic procedure encountered significant technical challenges: (1) severe tortuosity of the brachiocephalic trunk substantially impeded catheter manipulation, (2) only selective left coronary angiography could be successfully performed, and (3) right coronary artery visualization required a non-selective contrast injection through a pigtail catheter (Figure 1). Key angiographic findings included: Complete occlusion of the proximal left anterior descending artery (LAD) with developed collateral circulation; Critical 90% stenosis of the distal left circumflex artery (LCX) with preserved TIMI 3 flow; Suboptimal but patent right coronary artery (RCA) visualization. The procedure was ultimately discontinued following multidisciplinary consultation, based on: Resolution of ischemic symptoms; Prolonged procedural duration (Contrast agent volume: 300 ml; Radiation dose: 3,000 mSv.); Acceptable angiographic visualization of critical lesions.

On postoperative day 5, coronary computed tomography angiography (CTA) revealed the following vascular abnormalities: Complete occlusion of the mid left anterior descending artery (LAD) with established collateral circulation; Severe stenosis (≥90%) of the distal left circumflex artery (LCX); Non-significant stenosis (<50%) of the right coronary artery (RCA). Concurrent aortic CTA demonstrated characteristic postoperative anatomical alterations, including: Saccular protrusion of the aortic root; Crescent-shaped periaortic hypodensity, suggestive of either chronic dissection remnant or organized intramural hematoma (Figure 1). Following comprehensive cardiothoracic surgical consultation, the following determinations were made: Clear indication for coronary artery bypass grafting (CABG) based on angiographic findings; Contraindication for immediate surgical intervention due to extensive pleural adhesions from previous aortic surgery; Recommendation for referral to the original surgical center for specialized management. Detailed vascular access assessment identified: Prohibitive catheter navigation challenges via radial approach due to extreme vascular tortuosity; Significant risk of iatrogenic false lumen entry with femoral approach. Based on these findings, the patient was transferred to the original tertiary surgical institution for definitive management.

## 3 Discussion

### 3.1 Unique considerations in coronary assessment following aortic dissection surgery

This case illustrates three principal challenges in performing coronary interventions for post-aortic surgery patients: (1) procedural complexities arising from altered vascular anatomy, (2) limited vascular access alternatives, and (3) heightened difficulties in imaging interpretation. In the present case, marked tortuosity of the brachiocephalic trunk precluded optimal visualization of the right coronary artery. Subsequent coronary computed tomography angiography (CTA) findings suggested that a left-sided approach might have offered superior feasibility, thereby emphasizing the importance of comprehensive preprocedural vascular evaluation.

### 3.2 Optimal timing of imaging evaluation

This case highlights a critical clinical consideration: whether aortic computed tomography angiography (CTA) should be prioritized prior to emergency coronary angiography in post-aortic surgery patients presenting with suspected acute myocardial infarction. Existing evidence suggests that preoperative CTA significantly enhances procedural planning, with retrospective studies demonstrating a 2.1-fold improvement in surgical route selection accuracy (10). While time-sensitive management remains paramount in emergency settings, our experience suggests that rapid CTA acquisition in hemodynamically stable patients may provide indispensable anatomical guidance, thereby potentially averting procedure-related complications and optimizing interventional outcomes.

### 3.3 Optimization of long-term follow-up strategies

The delayed diagnosis of coronary occlusion in this case underscores the limitations inherent in current postoperative surveillance protocols. To address this clinical gap, we propose implementation of an integrated “aorto-coronary combined assessment” strategy, incorporating synchronous evaluation of both aortic and coronary anatomy via computed tomography angiography (CTA) during annual follow-up examinations. This approach is supported by level I evidence from the PROACT trial, which demonstrated a 42% relative risk reduction in major adverse cardiovascular events with systematic combined screening (11). The clinical rationale for this strategy lies in its capacity to: Facilitate early detection of progressive coronary artery disease; Enable timely initiation of intensive medical therapy; Permit elective revascularization when indicated. Such proactive surveillance may substantially mitigate the risk of acute ischemic events in this high-risk patient population.

### 3.4 Therapeutic dilemmas in complex cases

The incomplete revascularization achieved in this case exemplifies the inherent therapeutic challenges encountered in managing post-aortic surgery patients with complex coronary anatomy. Current clinical practice guidelines advocate for a multidisciplinary team-based approach (Class IIa recommendation) to optimize treatment decisions, necessitating close collaboration among cardiac surgeons, interventional cardiologists, and radiologists for comprehensive case evaluation (12). In selected cases, a hybrid surgical-interventional strategy may offer a clinically viable alternative, potentially balancing procedural risks with therapeutic benefits. This approach warrants particular consideration when: Conventional revascularization methods are anatomically prohibitive; Patient comorbidities preclude standard surgical interventions; High-risk anatomical features necessitate tailored solutions. The patient experienced no recurrence of chest pain prior to discharge, with complete normalization of cardiac biomarkers and heart function parameters. Following comprehensive aortic and coronary CTA evaluations, a thorough preoperative assessment was completed in preparation for subsequent surgical intervention.

## 4 Conclusion

The management of coronary artery disease in post-aortic surgery patients necessitates a highly individualized therapeutic approach, incorporating three principal strategies: Implementation of structured combined imaging surveillance protocols to facilitate early detection of both aortic and coronary vascular complications; Preprocedural anatomical assessment in acute settings, particularly through rapid computed tomography angiography, to optimize interventional planning and minimize procedural risks; Adoption of a multidisciplinary team-based management strategy, integrating expertise from cardiac surgery, interventional cardiology, and vascular radiology to determine optimal treatment pathways. This comprehensive approach addresses the unique anatomical and clinical challenges inherent in this high-risk patient population, potentially improving both short-term procedural outcomes and long-term prognostic results.

## Data Availability

The original contributions presented in the study are included in the article/Supplementary Material, further inquiries can be directed to the corresponding author.
